# Targeting CXCR4 reverts the suppressive activity of T-regulatory cells in renal cancer

**DOI:** 10.18632/oncotarget.20363

**Published:** 2017-08-19

**Authors:** Sara Santagata, Maria Napolitano, Crescenzo D'Alterio, Sonia Desicato, Salvatore Di Maro, Luciana Marinelli, Alessandra Fragale, Maria Buoncervello, Francesco Persico, Lucia Gabriele, Ettore Novellino, Nicola Longo, Sandro Pignata, Sisto Perdonà, Stefania Scala

**Affiliations:** ^1^ Functional Genomics, Istituto Nazionale per lo Studio e la Cura dei Tumori, Fondazione “G. Pascale”-IRCCS, 80131 Naples, Italy; ^2^ Uro-Gynecological Department, Istituto Nazionale per lo Studio e la Cura dei Tumori, Fondazione “G. Pascale”-IRCCS, 80131 Naples, Italy; ^3^ Department of Pharmacy, University of Naples Federico II, 80131 Naples, Italy; ^4^ Department of Hematology, Oncology and Molecular Medicine, Istituto Superiore di Sanità, 00161 Rome, Italy; ^5^ Urology Division, University Federico II, 80131 Naples, Italy

**Keywords:** CXCR4, immune suppression, renal cell carcinoma, T regulatory cells, tumor microenvironment

## Abstract

With the intent to identify biomarkers in renal cell carcinoma (RCC) the functional status of T-regulatory cells (Tregs) was investigated in primary RCC. Tregs were isolated from tumoral-(TT), peritumoral tissue-(PT) and peripheral blood-(PB) of 42 primary RCC patients and function evaluated through effector T cells (Teff) proliferation, cytokines release and demethylation of Treg Specific Region (TSDR). The highest value of Tregs was detected in TT with the uppermost amount of effector-Tregs-(CD4^+^CD25^hi^FOXP3^hi^CD45RA^-^). PB-RCC Tregs efficiently suppress Teff proliferation compared to healthy donor (HD)-Tregs and, at the intrapatient evaluation, TT-derived Tregs were the most suppressive. Higher demethylation TSDR was detected in TT- and PB-RCC Tregs vs HD-Tregs (*P* <0,001). CXCR4 is highly expressed on Tregs, thus we wished to modulate Tregs function through CXCR4 inhibition. CXCR4 antagonism, elicited by a new peptidic antagonist, Peptide-R29, efficiently reversed Tregs suppression of Teff proliferation. Thus Tregs functional evaluation precisely reflects Tregs status and may be a reliable biomarker of tumoral immune response. In addition, treatment with CXCR4 antagonist, impairing Tregs function, could improve the anticancer immune response, in combination with conventional therapy and/or immunotherapy such as checkpoints inhibitors.

## INTRODUCTION

Renal cell carcinomas (RCCs) is a disease with an estimated 338,000 new cases diagnosed worldwide [1] with approximately 30% of patients presenting with metastatic disease. High-dose IL-2 and Interferon-α were the principal therapies for metastatic RCC; due to the harsh-tolerance profiles and limited response rates, immunotherapies were replaced with targeted therapies but only everolimus has an impact on overall survival (NCCN guidelines Version 2.2016). The human IgG4 anti-PD-1 monoclonal antibody, Nivolumab, was recently approved to treat advanced (metastatic) clear cell renal cell carcinoma (ccRCC) patients who have received a prior anti-angiogenic therapy [2] resulting in 30% of clinical response. Immunotherapy efficacy is controlled by multiple mechanisms such as the recruitment of immunosuppressive cells to the tumor microenvironment (Tregs, Myeloid derived suppressor cells-MDSCs) [3]. T regulatory cells (Tregs) suppress a whole range of immune cells including B cells, NK cells, NKT cells, CD4^+^ or CD8^+^ T cells, monocytes and dendritic cells [4]. Nevertheless, Tregs role in cancer microenvironment is controversial: Tregs are associated with poor prognosis in renal [5], ovarian [6], pancreatic [7] and liver cancer [8], while others showed no correlation between tumor-infiltrating Tregs and patient outcome [9]. Tregs are identified through the surface expression of CD3, CD4, CD25, FOXP3, CD127 and CD45RA. In addition ICOS, a member of CD28/CTLA-4 family and CD39/ENTPD1 defines a highly suppressive Treg population [10]. CTLA-4 and PD-1 have also been proposed as key molecules in generation and/or suppressive function of Tregs [11]. Cancer patients derived Tregs usually express a distinct profile of chemokine receptors, such as CCR4, CXCR4 and CCR5, which facilitates their migration into tumors in response to the corresponding chemokine ligands derived from tumor microenvironment [12]. With the intent to characterize the local antitumor immune response Tregs were functionally evaluated in 42 RCC patients. Moreover, CXCR4 antagonism was investigated as a strategy to impair Treg efficiency.

## RESULTS

### Tregs isolated from primary tumor (TT), peritumoral tissue (PT) and peripheral blood (PB) of renal cancer (RCC) patients are highly activated

**Figure 1:**
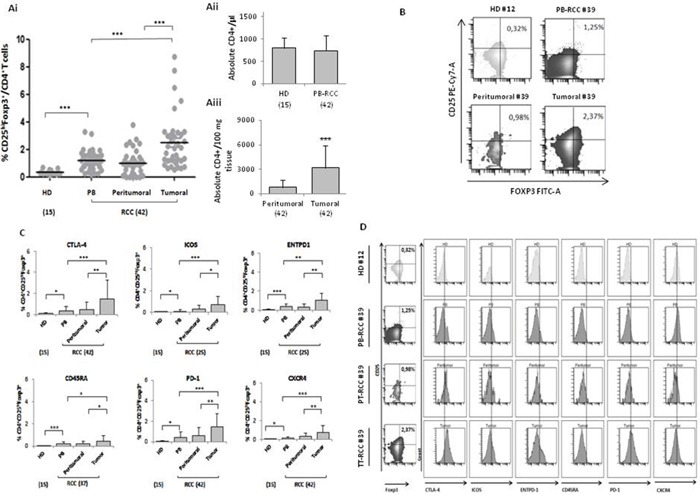
High CD4^+^CD25^hi^Foxp3^+^ Tregs in RCC tumors **(Ai)** Percentage of CD4^+^CD25^hi^Foxp3^+^ (15 HD vs 42 PB p<0.001; HD vs 42 peritumoral *P* <0.05; HD vs 42 tumor *P* <0.001) (tumor vs peritumoral *P* <0.001; tumor vs PB *P* <0.001). **(Aii)** Absolute number of CD4+ in 15 HD, 42 peripheral and (Aiii) 42 tumor/peritumor tissue. **(B)** Representative example in HD (#12) (absolute CD4+/μl: 722) and RCC patients (# 39) (absolute CD4+/μl in PB:733; absolute CD4+/100 mg tissue: peritumoral 850 vs tumoral 2600). **(C)** Percentage of CTLA-4, PD1, CXCR4 ICOS, ENTPD1 and CD45RA, in CD4^+^CD25^hi^Foxp3^+^ cells. **(D)** Representative plots of Tregs from HD (#12) and RCC-PB, -PT and -TT patient (#39).

**Figure 2:**
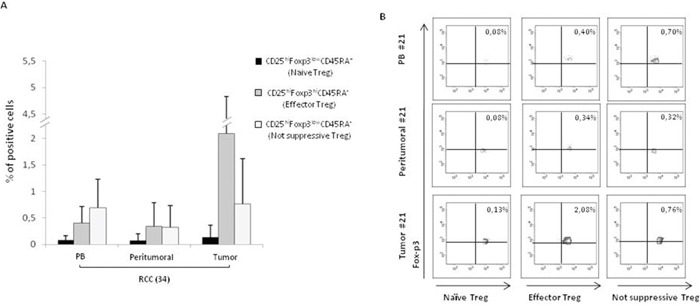
Higher CD25^hi^Foxp3^hi^CD45RA^-^ effector Tregs in RCC tumors **(A)** Phenotypic characterization of naïve, effector and not suppressive Tregs in 34 RCC patients (PB and PT vs TT, *P* <0,001). **(B)** Representative analysis of Tregs subpopulations (patient #21).

**Figure 3:**
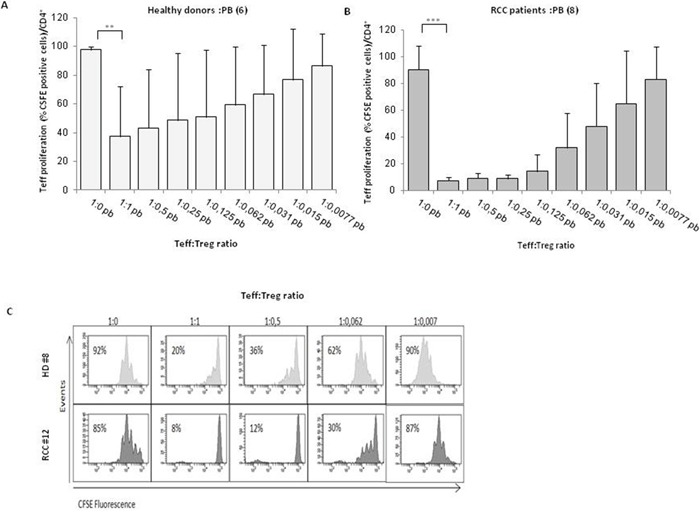
PB-Tregs from RCC patients are more suppressive than HD-Tregs **(A-B)** AutologousCFSE-labeledCD4^+^CD25^-^ T cells were co-cultured with CD4^+^CD25^+^ isolated from peripheral blood of 6 HD and 8 RCC patients (at the Teff:Treg ratios from 1:1 to 1:0.007; the 1:0 ratio indicated the positive control). After 5 days of stimulation with Dynabeads Human T-Activator CD3/CD28, CFSE^+^CD4^+^ T cells were analyzed for their proliferation by CFSE dilution. **(C)** Representative analysis of CFSE-labeled Teff proliferation of HD (#8) and RCC (#12) patient in the presence of Tregs.

Tregs, identified as percentage of CD4^+^CD25^hi^FOXP3^+^ cells, were evaluated in 42 consecutive renal cancer patients (Table 1). A significantly higher number of Tregs was detected in 42 RCC-PB as compared to 15 healthy donors (HD) (Figure 1Ai) Intrapatients analysis demonstrated higher Tregs in TT versus PT and PB. In Figure 1B a representative plot of HD-Tregs and RCC-Tregs was shown. As shown in Figure 1C-1D, TT-Tregs displayed the highest level of CTLA-4, ICOS, ENTPD1, CD45RA, PD-1 and CXCR4. According to the Tregs functional classification that distinguish CD25^hi^FOXP3^hi^CD45RA^-^ as effector and CD25^hi^FOXP3^low^CD45RA^-^ as not suppressive Tregs [13], the highest number of effector Tregs was detected in TT (PB and PT vs TT, *P* <0,001) (Figure 2A); in Figure 2B a representative analysis of Tregs subpopulations is shown (PB/PT/TT). PB-Tregs from RCC patients cocultured with autologous Teff cells more efficiently suppress Teff proliferation compared to HD-Tregs (Figure 3A-3B). In Figure 3C a representative analysis of CFSE-labeled Teff proliferation-Treg dependent was shown. As control, anti-CD3/CD28-stimulated CD4^+^CD25^+^ T cells were anergic while Teff intensively proliferated (Supplementary Figure 1).

**Table 1:** Clinical characteristics of RCC patients

No. of patients	42
Median age (range), yr	59 (28-83)
Gender (Female/Male)	14/28
Pathological stage (pT1/pT2/pT3/pT4/missing)	27/4/9/1/1
Tumor size cm( >5/≤5/missing)	14/27/1
Histologic variant(clear cell/papillary/chromophobe/papillary-clear cell/mucinous tubular and spindle cell)	31/4/2/4/1
Furhman's grade (1/2/3/missing)	3/11/23/5

### TT-Tregs are more suppressive than PB- and PT- Tregs in RCC patients

**Figure 4:**
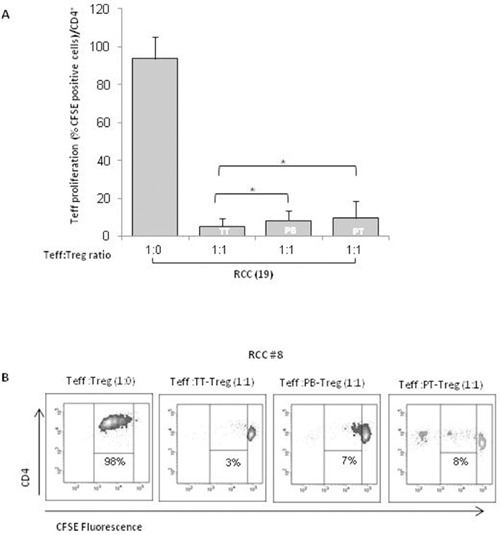
TT-Tregs are more suppressive than PB and PT-Tregs in RCC patients **(A)** AutologousCFSE-labeled CD4^+^CD25^-^ T cells were co-cultured with CD4^+^CD25^+^ isolated from peripheral blood (PB), tumor (TT) and peritumoral (PT) of 19 RCC patients as indicated. Histograms show the results relatively to 19 RCC patients. **(B)** Representative FACS analysis of suppression assay with PB-Tregs RCC isolated Tregs in one RCC patient (#8).

**Figure 5:**
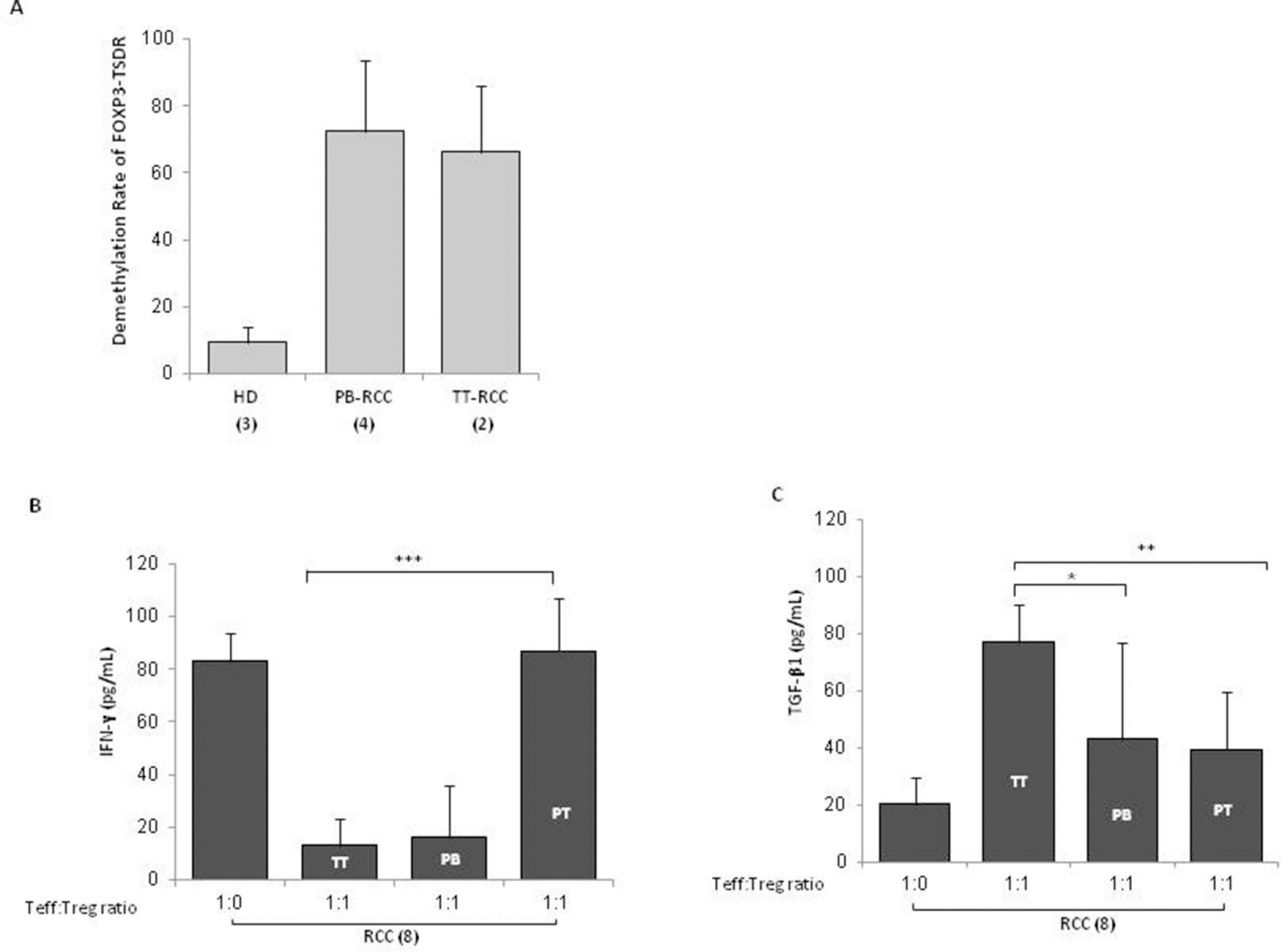
TT-Tregs are more suppressive than PB and PT-Tregs in RCC patients **(A)** Demethylation rate of *FOXP3*-TSDR in TT-Tregs and PB-Tregs versus HD-Tregs (2 TT-Tregs vs 4 PB-Tregs vs 3 HD respectively). **(B)** IFN-γ-Treg dependent ELISA and (C) TGF-β1-Treg dependent ELISA from 8 RCC patients.

In Figure 4A, TT-, PT- and PB- isolated Tregs significantly suppressed autologous Teff cells proliferation (*P* <0,001). In particular, TT-Tregs more efficiently suppressed T-effector proliferation compared to PT- and PB-Tregs (Figure 4A). In Figure 4B a representative suppression assay was shown. Treg function is also regulated through the status of methylation of Treg-specific demethylated region (TSDR). Thus *FOXP3*-TSDR demethylation status was investigated on TT- and PB-Tregs from RCC patients and compared to HD. High demethylation rate was detected in TT-RCC and PB-RCC vs HD derived Treg cells (*P* <0,001) (Figure 5A). As reference sequence the methylation of CpG sequences of IFN transcription regulatory factors 8 (IRF8) was considered [14, 15]. Moreover to evaluate Tregs function culture supernatants were evaluated for IFN-γ and TGF-β1 on day 5 of cocolture. As shown in Figure 5B, a significant decrease of IFN-γ was observed when TT-Tregs were added to autologous Teff cells. Of interest, very low IFN-γ production was observed in cultures with PB-Tregs. Surprisingly, a dramatic increase in IFN-γ was observed in coculture of PT-Tregs. This increase could be ascribed to IFN-γ production from tumor infiltrating lymphocytes (TIL) that induce inhibitory T cell ligands such as PD-L1 [16]. As expected, a significant increase of TGF-β1 was observed when TT-Tregs were added to autologous Teff cells (Figure 5C). TGF-β1 and IL-10 mRNA expression was also evaluated in TT and PT RCC tissues. Consistent with an increased Treg function, TGF-β1 expression, though not significant, increased in tumoral tissues (PT vs TT: 0.06±0.08 vs 0.20±0.30) while IL-10 expression dramatically increased in TT as compared to PT samples (tumoral vs peritumoral: 0.36±0.37 vs 0.08±0.14, p<0,01) (Supplementary Figure 2). Altogether these data suggest that, although TT-Tregs share some phenotypic similarities with both PT- and PB-Tregs, they are functionally more suppressive.

### CXCR4 antagonists impair RCC-tregs function

**Figure 6:**
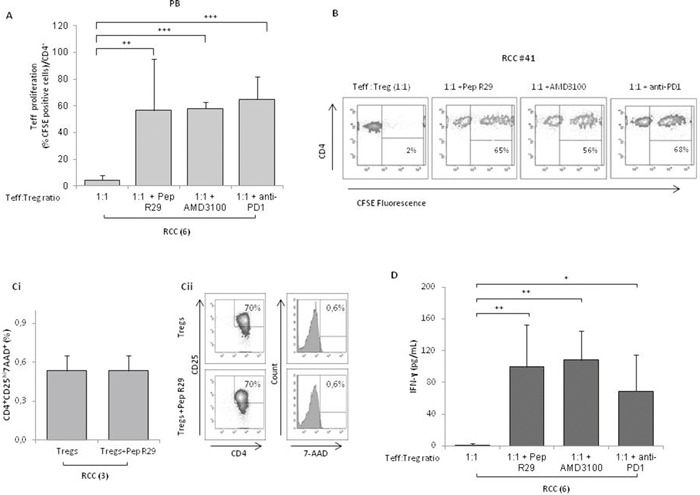
CXCR4 antagonists reverses the suppressive capability of Tregs from RCC patients **(A)** CFSE-labeledCD4^+^CD25^-^ T cells were co-cultured at the 1:1 Teff:Treg ratio with CD4^+^CD25^+^ PB Tregs from 6 RCC patients. Tregs were pretreated for 30’ at 37°C in 5% CO_2_ with R29 (10 μM), AMD3100 (10 μM) or anti-PD1 (20 μg/mL) and then added to the Teff cells for 5 days. Teff cells were analyzed for their proliferation by CFSE dilution. **(B)** Representative effect of CXCR4 antagonists (R29, AMD3100) or anti-PD1 on Teff-CSFE proliferation-Treg mediated in RCC patients (#41). **(Ci)** Viability of isolated Tregs from 3 RCC patients pretreated for 30’ at 37°C in 5% CO_2_ with R29 (10 μM) washed and then stained with 7-AAD. **(Cii)** Rapresentative analysis of 7-AAD staining in Tregs isolated from one RCC patients. **(D)** IFN-γ-Treg dependent production through ELISA from 6 RCC patients with Tregs pretreated for 30’ at 37°C in 5% CO_2_ in presence of R29 (10 μM), AMD3100 (10 μM) or anti-PD1 (20 μg/mL).

Since CXCR4 is highly expressed by Tregs, we speculate that interfering with CXCR4 signaling might inhibit the Tregs suppressive function. A new family of CXCR4 antagonists was recently developed by us [17]. In Figure 6A the effect of the canonical CXCR4 antagonist AMD3100 and the new Peptide R29, was evaluated [17, 18]; when PB-Tregs were pretreated with CXCR4 antagonists and then added to coculture with Teff, an efficient reversal of Treg suppressive CSFE proliferation was detected (1:1 vs 1:1+ Peptide R29: 5±5% vs 57±39%; 1:1 vs 1:1+AMD3100: 5±5% vs 58±5%). As positive control, pretreatment of Tregs with anti-PD1 reverted PB-RCC derived Treg suppression of Teff proliferation at a comparable extent of Peptide R29 (Figure 6A). In Figure 6B a representative example of reversal of Treg suppressive capability was shown. Treatment of Teff with AMD3100 or R29, in absence of Tregs, did not affect proliferation (Supplementary Figure 3A and 3B). No significant effect of CXCR4 antagonism was observed on Teff proliferation in presence of HD isolated Tregs (Supplementary Figure 3C). Comparable results were obtained from TT isolated Tregs (data not shown). To exclude a toxic effect of CXCR4 antagonist on Tregs, viability was evaluated on Tregs pretreated with Pep R29 through the 7-AAD exclusion test; as shown in Figure 6Ci-6Cii, when PB-Tregs were incubated with Pep R29 viability was unaffected. Moreover, a significant increase of IFN-γ was observed when PB-Tregs were pretreated with Pep R29, AMD3100 and anti-PD1 and then added to autologous Teff cells (1:1 vs 1:1+ Peptide R29: 1±2 vs 99±53; 1:1 vs 1:1+AMD3100: 1±2 vs 108±36; 1:1 vs 1:1+ anti-PD1: 1±2 vs 70±45) (Figure 6D) suggesting a recover in T-effector proliferative activity. As control, supernatant from isolated Teff plus and minus Dynabeads Human T-Activator CD3/CD28 were evaluated for the IFN-γ secretion (Supplementary Figure 4).

## DISCUSSION

Tumors actively escape the immune system through complex mechanisms including inhibitory receptors, secreted soluble inhibitors (IL-10 and TGF-β) and Tregs [19]. In the present study Tregs were evaluated in 42 RCC patients: higher number of Tregs was detected in tumoral tissue as compared to peritumoral and peripheral Tregs as previously described in patients with head and neck squamous cell carcinoma, breast, pancreatic, stomach and liver cancer [20]. In here, Tregs expressing CTLA-4, ICOS, ENTPD1, CD45RA and PD-1 were the majority among tumoral Tregs and the so called effector Tregs, CD25^hi^FOXP3^hi^CD45RA^-^ highly suppressive cells, were the most represented among tumor Tregs tissue derived [13]. Higher number of intratumoral Tregs with activated phenotype was previously reported in non-small-cell lung cancer patients and rectal cancer [21]. The elevated number of Tregs in RCC environment raises the question of their immunosuppressive capability. We found that tumoral Tregs display higher immunosuppressive activity compared to peritumoral and peripheral Tregs as demonstrated by reduced Teff proliferation and IFN-γ production. The high IFN-γ production in the cocolture of peritumoral Treg and T effectors can probably be ascribed to IFN-γ production from TIL cells that induce inhibitory T cell ligands such as PD-L1 [16]. Moreover, high Treg activity was supported by TGF-β production in culture supernatants [10]. These data suggest that functional Tregs accumulated in the RCC tumors control tumor immunity. Tregs function is regulated through methylation of Treg-specific demethylated region (TSDR). TSDR is a CpG dinucleotide rich domain and highly conserved region within the conserved non coding sequences 2 (CNS2), located in the first intron of *FOXP3* gene [22]. TSDR-hypermetilated Tregs impaired suppressive function [23]; recent evidence demonstrated TSDR demethylation in liver biopsy and peripheral blood samples from patients with advanced grades of HCC compared to control subjects with nonmalignant disease [24]. Herein we show that TSDR demethylation increases in RCC isolated Tregs suggesting a higher suppressive function compared to healthy donors isolated Tregs. These results were compared to the demethylation induced by DNMTi azacytidine (AZA) and IFN-γ on unrelated sequence in the promoter of the Interferon Regulated Factor IRF-8 [14, 15]. Tregs from RCC patients express higher CXCR4 compared with Tregs from healthy donors. CXCR4 receptor is activated by the ligand CXCL12 that regulates T cell access and recruits immunosuppressive population to tumoral microenvironment including interleukin-10–producing plasmacytoid dendritic cells, Tregs and MDSCs. In a mouse model of ovarian cancer the CXCR4 antagonist, AMD3100, determined several anti-tumor effects including increased tumor cell death, reduced dissemination and better survival of the treated animals. Significantly, it was observed a selective reduction in the recruitment of Foxp3^+^ T cells in comparison with CD8^+^ T cells [25]. Blockade of both CXCR4 and PD-1 prevents suppression of immune cell function in HCC tumors, enhances immune cell tumor penetration and activation, and ultimately delays HCC progression [26]. In our experience CXCR4 antagonism with AMD3100 or with a new class of CXCR4 antagonists (Peptide R29) reverts Treg suppressive function in RCC patients. CXCR4 transduction may regulate some genes critical in Treg function since CXCL12/CXCR4 signaling plays a key role in human thymocyte development, promoting survival and expansion of human early T-cell progenitors [27].

The mechanism through which CXCR4 antagonists impact on Treg function is under evaluation. A possibility is that CXCR4 transduction impairs the methylation status of *FOXP3*. In metastatic renal carcinoma IL-2 based immunotherapy leads to substantial expansion of T-cells with a demethylated TSDR. Preliminary evidence displayed a reduced demethylation in TSDR when RCC derived Tregs were treated with CXCR4 antagonists (Santagata et al, manuscript in preparation). Post translational modification such as acetylation can regulate Treg-FOXP3 activity [28]. Because CXCR4 signaling transduces also on FOXP3 and HDACis upregulated CXCR4 mRNA expression [29], it is possible that CXCR4 modulation affects the acetylation status of *FOXP3* gene promoter.

Although the anti PD-1 Nivolumab was recently approved to treat advanced (metastatic) clear cell renal cell carcinoma (ccRCC), only a minority of patients display a durable anti PD-1 response. This effect might be due to the tumor microenvironment lacking the efficient T-effector cells access to tumors and to a recruitment of immunosuppressive cells. Targeting the CXCR4-CXCL12 axis could revert the tolerogenic polarization of the microenvironment rich of immunosuppressive cells such as Tregs cells M2 and N2 neutrophils [25, 30, 26], improving immunotherapeutic intervention in patient with RCC. Preliminary evidence from an ongoing observational trial demonstrates that Tregs functional status in peripheral blood of nivolumab treated metastatic renal cancer patients is predictive of drug sensitivity [31]. Although the number of Tregs isolated from peripheral blood was adequate to functionally evaluate the role of Tregs in RCC patients, the Tregs isolated from primary tumors was a limiting factor. Moreover the interpatients variability required an adequate number of patients to draw conclusions. In conclusion RCC patients at peripheral, tumor and peritumoral sites, contain high numbers of functional Tregs and intratumoral Tregs actively limits the tumoral immune response. Thus monitoring Treg function also in peripheral blood could be informative on the antitumor immune response and possibly predictive of immunotherapy response. Moreover, CXCR4 antagonism is able to reverse *in vitro* Tregs suppressive capability on effector T cells proliferation suggesting that targeting CXCR4 may improve immunotherapy in RCC.

## MATERIALS AND METHODS

### Patients and specimens

RCC samples were collected from 42 RCC patients undergone partial/radical nephrectomy at the Urology Unit of Istituto Nazionale per lo Studio e la Cura dei Tumori, Fondazione “G. Pascale” and Genitourinary Oncology and Rare Cancer Center, Federico II University in Naples. Renal tumor (TT) and peritumoral tissue (PT) specimens were collected at the time of surgery.

The distance 1cm was the minimal distance between tumor and *normal*-appearing renal *tissue sampled (PT)*. In addition, 8-mL of heparinized peripheral blood (PB) was collected from each patient before surgery. Heparinized blood was also collected from 15 healthy donors (HD). Patients features are shown in Table 1. The research protocol was approved by Human Ethical Committee of Institute (n. CEI/423/13).

### Cells preparation

Peripheral blood samples from cancer patients and healthy donors were drawn into heparinized tubes (BD Biosciences) and centrifuged on Ficoll–Hypaque gradients (GE Healthcare Bioscience). For tissue-infiltrating lymphocytes isolation, freshly isolated tumors sample and corresponding peritumoral renal tissue biopsies were minced into small pieces, digested with 1 mg/mL of collagenase (Sigma-Aldrich) for 30 minutes at 37°C, then transferred to a cell strainer (70μm Nylon) (BD Biosciences) and gently separated by using a syringe plunge. The collected cells underwent to Ficoll–Hypaque gradient centrifugation, washed and immediately used for experiments.

### Antibodies and flow cytometric analysis

Flow cytometry was performed on venous peripheral blood collected in heparin-coated vacutainer tubes, on tumor and peritumoral tissues using a FACSCanto II 6-colour flow cytometer, daily calibrated with calibrite beads (Fitc, Pe, PerCP and APC) and compbeads (Pe-Cy7 and APC-Cy7; Becton Dickinson, San Jose, CA, USA). Fluorochrome-labelled monoclonal antibodies (BD Bioscience) for identification of circulating and tissue Treg cells were used: Fitc-anti-FOXP3, Pe-Cy7-anti-CD25, APC-Cy7-anti-CD4, APC-anti-CD45RA, Pe-anti-CD152 (CTLA-4), PercP-anti-CD184 (CXCR4), APC-anti-CD279 (PD-1), Pe-anti-CD278 (ICOS) and APC-anti-CD39 (ENTPD1). Intracellular staining for FoxP3, ICOS and CTLA-4 was performed using a commercially available kit (BD Cytofix/Cytoperm; fixation and permeabilization kit; BD Pharmingen) according to the manufacturer's instructions. A minimum of 100.000 events for each sample were collected and data were analysed using FacsDiva software 6.1.3 (BD Bioscience). The absolute number of CD4 was calculated as follow: [total withe blood cell count (cells/uL) x percent CD4]/100 or [total tumor-infiltrating immune cell count (cells/100 mg tumor) x percent CD4]/100.

### Purification of T cell subsets

Peripheral, tumor and peritumoral CD4^+^CD25^+^ Tregs and peripheral CD4^+^CD25^−^ T effector (Teff) cells were isolated using the Dynabeads Regulatory CD4^+^CD25^+^ T cell kit. Briefly, CD4^+^ cells were separated by negative selection, using the antibody mix human CD4. In the second step, a depletion beads solution was added to remove the non-CD4 cells. Then, CD25-beads was added to CD4^+^ T cells to capture the CD4^+^CD25^+^ Tregs and the remaining fraction was used as CD4^+^CD25^−^ Teff cells. Finally, Dynabeads CD25 was removed from the cells. All purification steps were performed according to the manufacturer's instructions (Invitrogen by Life Technologies) and collected cells were found to be > 95% pure by flow cytometry.

### Suppression assay

Carboxyfluorescein diacetate succinimidyl ester (CFSE)-labeled autologous CD4^+^CD25^−^ T cells from peripheral blood (CellTrace CFSE Cell Proliferation Kit, Molecular Probes, by Life Technologies) were cultured with peripheral CD4^+^CD25^+^ Tregs at different ratios (1:1, 1:0.5, 1:0.25, 1:0.125, 1:0.06, 1:0.03, 1:0.015, 1:0.007 and 1:0 respectively) and with tumor or peritumoral CD4^+^CD25^+^ Tregs in 1:1 ratio. Cells were cultured (5×10^3^ cells/well) in U-bottom 96-well plates with RPMI medium (Thermo scientific, HyClone Laboratories, Inc) supplemented with 2-mM L-glutamine, 100 U/ml penicillin, 100 μg streptomycin, and 10% fetal bovine serum. Cells were stimulated for 5 days in the presence of Dynabeads Human T-Activator CD3/CD28 (Gibco by Life Technologies). As additional control, Teff cells were cultured alone with and without the Dynabeads Human T-Activator CD3/CD28 and Tregs were cultured alone with Dynabeads Human T-Activator CD3/CD28. The suppressive activity of Tregs was assessed by evaluating the CFSE-labeled Teff cells by FACS analysis. Furthermore, Tregs were pretreated for 30’ at 37°C in 5% CO_2_ with 10 μM of Peptide R29, a modified Peptide R (also known as compound 10) a new class of CXCR4 antagonists [17, 18], 10 μM of AMD3100 (Sigma-Aldrich) a selective CXCR4 antagonist or 20 μg/mL of anti PD-1 before coculture with Teff cells. As control, activated Teff cells were cultured in the presence of CXCR4 antagonists.

### Methylation studies: genomic DNA isolation, bisulfite conversion, and qPCR

Genomic DNA (gDNA) of peripheral Tregs isolated from 4 HD and 5 RCC patients was obtained by using a traditional phenol/chloroform extraction method with minor modifications. Bisulfite treatment of 500 ng genomic DNA was performed by using the EZ DNA Methylation™ Kit (ZYMO Research) according to the manufacturer's instructions. qPCR was prepared by using SensiMix SYBR Kit (Bioline, London) and performed by LightCycler^®^ 480 System (Roche Diagnostics). Primers for methylation and demethylation-specific FOXP3-TSDR and computing of the demethylation rate (DMR) of *FOXP3*-TSDR were previously described [12]. Briefly, we used the following formula: 100/[1 + 2(CtTG- CtCG)] × 100%, where CtTG represents the cycle threshold (Ct) achieved with TG (demethylated) primers and CtCG represents the Ct achieved with CG (methylated) primers. For female patients, this rate was corrected by a factor of 2 because one of the two TSDR alleles is methylated as a result of X inactivation.

### Cytokine assay

IFN-γ and TGF-β1 were measured by ELISA assay on the culture supernatant collected on day 5 from suppression experiments. In particular, cytokine concentration was assessed by Human IFN-gamma Instant ELISA (Bender MedSystems) and Human TGF-β1 ELISA kit (Boster Biological Technology Co). Samples were acquired by LB 940 Multimode Reader Mithras (Berthold Technologies).

### Viability assays

Isolated CD4^+^CD25^+^ T cells were incubated for 30’ at 37°C in 5% CO_2_ with or without 10 μM of Peptide R29. After 30’ the cells were washed and then stained with Fitc-anti-CD25, APC-Cy7-anti-CD4 (BD Bioscience) and 7-amino-actinomycin D (7-AAD) (BioLegend) and analyzed by flow cytometer.

### Real-time PCR

RNA was extracted from frozen RCC tumor and corresponding peritumoral tissues using the RNeasy Mini kit (Qiagen), according to manufacturer's instructions. The primer sequences were designed using Primer3: *GUSB*, sense 5’-AGCCAGTTCCTCATCAATGG-3’; antisense 5’-GGTAGTGGCTGGTACGGAAA-3’; *IL10*, sense 5’-TGGGGGAGAACCTGAAGA -3’; antisense 5’-TGGCTTTGTAGATGCCTTTC-3’; *TGFB1*, sense 5’- ATGGGGGCTGTATTTAAGGA-3’; antisense 5’-AGGCAGAGAGGGAGAGAGA -3’. Samples were run in triplicate, and their relative expression was calculated in the following formula using *GUSB* as endogenous control: 2^-ΔΔCt^.

### Statistical analysis

Data were presented as mean values ± s.d.; statistical analyses were done using the paired and unpaired two-tailed Student's *t-* test. *P*-values less than 0.05 were considered statistically significant (*P< 0.05; **P< 0.01; ***P< 0.001).

## SUPPLEMENTARY MATERIALS AND FIGURES



## Abbreviations

RCCs: renal cell carcinomas
ccRCC: clear cell renal cell carcinoma
Tregs: T regulatory cells
ICOS: inducibile T-cell costimulator
CD39/ENTPD1: ectonucleoside triphosphate diphosphohydrolase 1
ICRs: immune-checkpoint receptors
TSDR: Treg-specific demethylated region
CNS2: conserved non coding sequences 2
TT: tumor tissue
PT: peritumoral tissue
PB: peripheral blood
HD: healthy donors
Teff: T effector cells
CFSE: carboxyfluorescein diacetate succinimidyl ester
DMR: demethylation rate
Ct: cycle threshold
7-AAD: 7-amino-actinomycin D
AZA: azacytidine
