# Supplementary material for: Targeting CXCR4 reverts the suppressive activity of T-regulatory cells in renal cancer
Source: Oncotarget. 2017 Aug 19;8(44):77110–20. doi: 10.18632/oncotarget.20363 (PMC5652768; doi:10.18632/oncotarget.20363)
Supplement: Supplementary file 1 [file oncotarget-08-77110-s001.pdf]

Targeting CXCR4 reverts the suppressive activity of T-regulatory cells in renal cancer

SUPPLEMENTARY MATERIALS

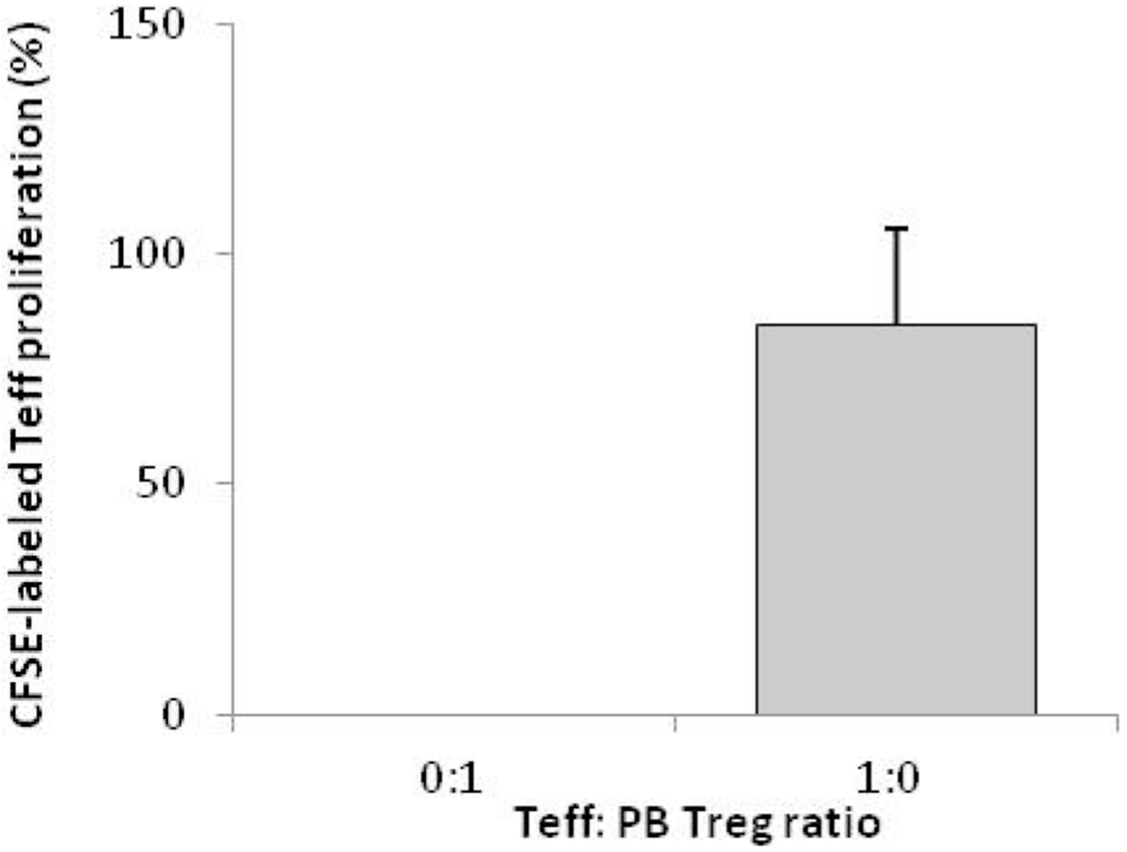

Supplementary Figure 1: Effect of anti-CD3/CD28 stimulation on RCC-$CD4^+CD25^+$ T and $CD4^+CD25^-$ T cells proliferation: anti-CD3/CD28-stimulated $CD4^+CD25^+$ T cells were anergic while Teff intensively proliferated.

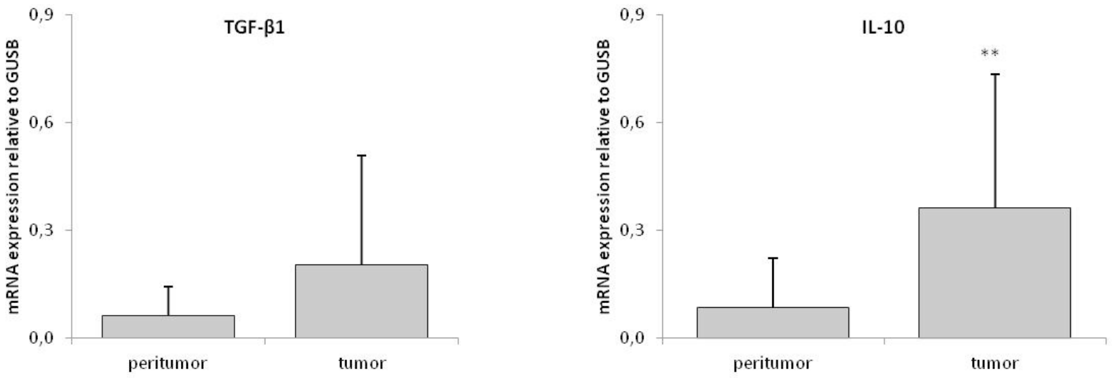

Supplementary Figure 2: Measurement of TGF-β1 and IL-10 mRNA expression by real-time PCR in tumor and corresponding peritumoral tissues. Relative mRNA expression was calculated according to $2^{-\Delta\Delta Ct}$ method.

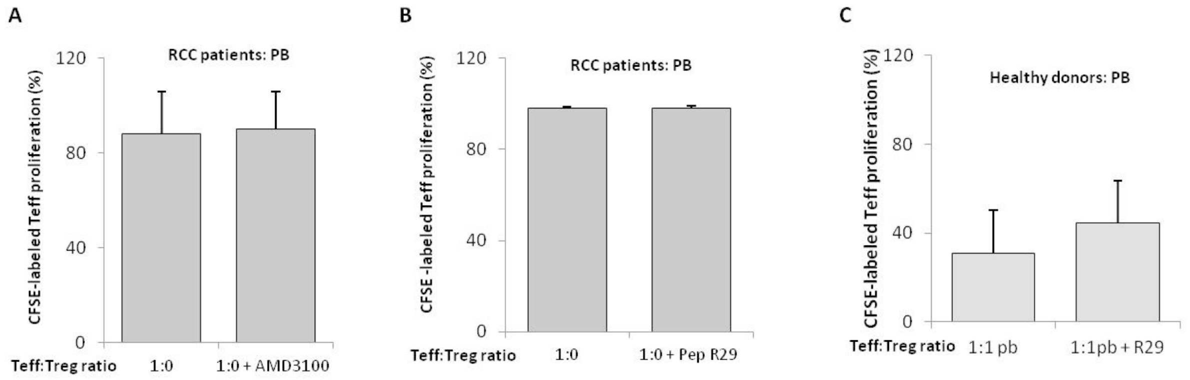

Supplementary Figure 3: Effect of CXCR4 antagonism on CFSE-labeled Teff proliferation alone in absence or presence of AMD3100 **(A)** and Peptide R29 **(B)**. **(C)** CFSE-labeled$CD4^+CD25^-$ T cells were co-cultured at the 1:1 Teff:Treg ratios with $CD4^+CD25^+$ isolated from peripheral blood of healthy donors; Treg cells were pretreated with or without Peptide R29 and after 5 days of stimulation with anti-CD3/CD28, $CFSE^+CD4^+$ T cells were analyzed for their proliferation by CFSE dilution.

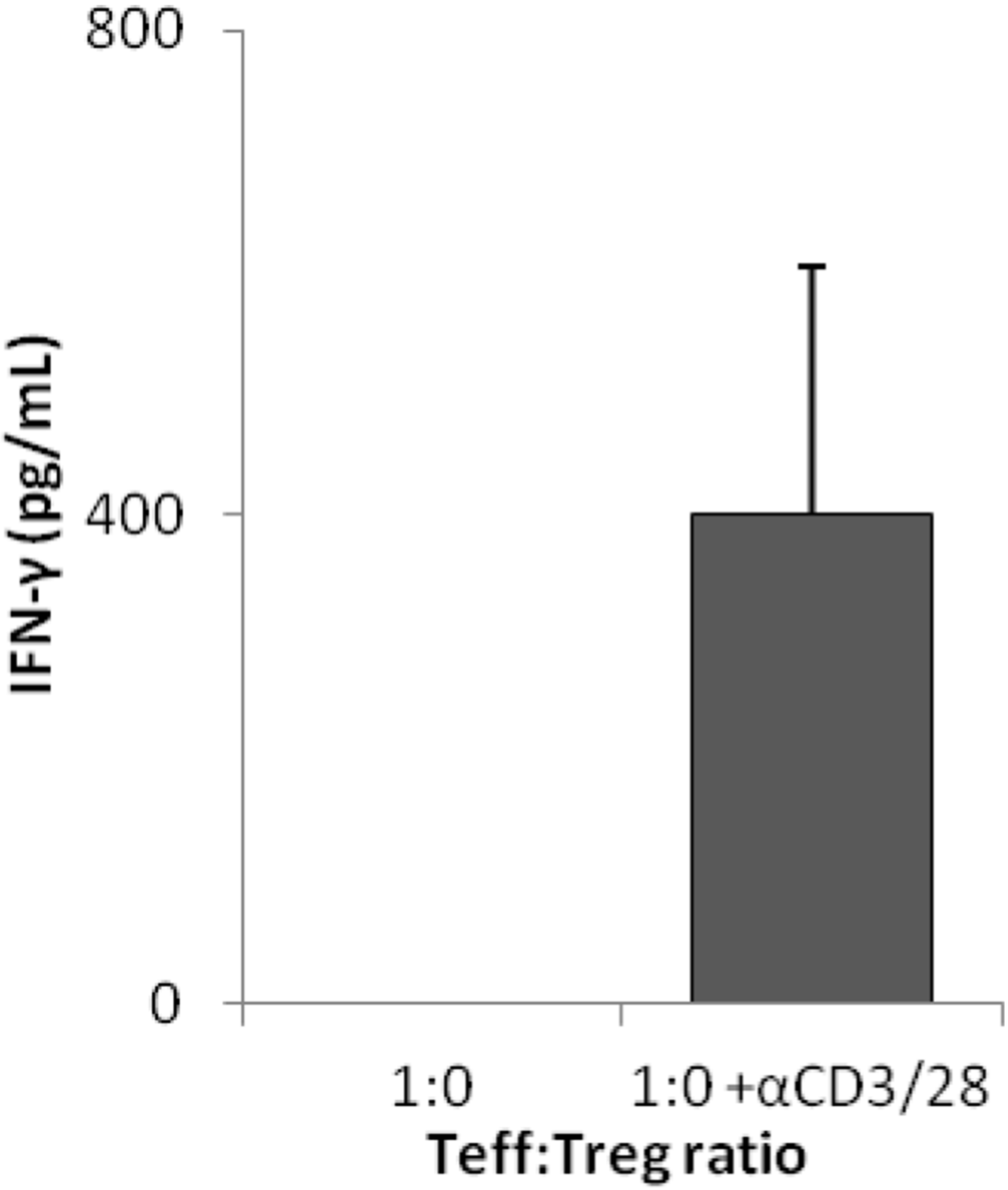

Supplementary Figure 4: IFN-γ-Treg dependent production through ELISA in culture supernatant from 6 RCC patients. Isolated Teff cells were stimulated for 5 days with or without Dynabeads Human T-Activator CD3/CD28.
